# What do healthcare workers need? A qualitative study on support strategies to protect mental health of healthcare workers during the SARS-CoV-2 pandemic

**DOI:** 10.1186/s12888-023-04686-z

**Published:** 2023-03-24

**Authors:** Theresa Halms, Martina Strasser, Irina Papazova, Philipp Reicherts, Giulia Zerbini, Svenja Grundey, Esther Täumer, Manuela Ohmer-Kluge, Miriam Kunz, Alkomiet Hasan

**Affiliations:** 1 Department of Psychiatry and Psychotherapy, Medical Faculty, University of Augsburg, Bezirkskrankenhaus, Augsburg, Germany; 2 Department of Medical Psychology and Sociology, Medical Faculty, University of Augsburg, Augsburg, Germany; 3 Pestalozzigymnasium München, Munich, Germany; 4 Department of Psychiatry and Psychotherapy, University Hospital, Klinikum der Universität München, Ludwig-Maximilians University Munich, Munich, Germany; 5 University Medical Centre Augsburg, Augsburg, Germany

**Keywords:** Healthcare workers, COVID-19, SARS-CoV-2, Mental health, Barriers, Psychological support

## Abstract

**Background:**

To support healthcare workers (HCWs) during the increased burden caused by the SARS-CoV-2 pandemic, numerous recommendations for action and possible interventions have been developed. However, the actual protective factors, needs and desires of those affected, as well as potential barriers to implementing psychological interventions, have been insufficiently examined. This study addresses this research gap and captures HCWs’ experiences and views.

**Methods:**

Medical personnel including nursing staff and physicians were recruited for a qualitative study regarding protective factors as well as barriers to the implementation of support services. We conducted 21 individual, semi-structured interviews with members of the medical staff at tertiary care center in Germany. The collected data were analyzed using a qualitative content analysis.

**Results:**

The analyses showed that social interaction in the professional and private context was rated as helpful in coping with the challenges of everyday work amplified by the SARS-CoV-2 pandemic. The results also suggest that the available support services, despite being highly valued, were rarely accessed. Possible barriers included the fear of negative consequences when asking for support. It could be deduced that the fear and shame of admitting one’s own mistakes as well as the work-related tradition of showing no weakness could be the underlying factors for this fear.

**Results:**

The results of this study suggest that medical staff need a more extensive range of low-threshold support services, which should be adapted to the respective needs of the professional groups. The study also provides indications that the norms and expectations represented in the hospital system require critical reflection.

## Background

The SARS-CoV-2 pandemic has confronted healthcare workers (HCWs) worldwide with an unprecedented challenge. Apart from the obvious risk of infection, equipment shortages, high numbers of patients as well as high mortality rates and strict infection prevention and control measures, amongst other factors, have placed an enormous mental strain on HCWs [1–3].

These multifaceted stressors have affected HCWs’ mental health and well-being. A systematic review including 33 studies investigating the prevalence of mental health problems in HCWs showed a significant increase in the prevalence of stress, depression, anxiety symptoms and a decrease in sleep quality among HCWs during the SARS-CoV-2 pandemic [4]. Among German HCWs, one online survey conducted during the first wave of the pandemic reported that 17–22% of HCWs met the criteria for clinically significant levels of depression and 17–19% for anxiety [5]. Further, the findings indicated an association between a higher prevalence of depressive symptoms and diminished trust in colleagues, insufficient compensatory time-off and increased alcohol consumption [5]. Moreover, one survey from our group showed that while job strain and uncertainty about the future were the most frequently reported causes for burden among HCWs, psychosocial support and leisure time were found to be important resources [6]. Additionally, resources and coping strategies that strengthen HCWs’ resilience, including support from family as well as professional support and sufficient provision of personal protective equipment (PPE), have been found to play an important role [7]. One systematic review including 31 studies found that increased psychological resilience positively affected HCWs’ mental health and was associated with a lower incidence of anxiety, stress and insomnia related to the pandemic [8]. The results further showed that psychological interventions, such as meditation therapy or coping skills training, as well as effective leadership, organizational support, adequate PPE and the provision of timely information were the most common strategies to promote resilience [8]. Just recently, we showed that in addition to psychosocial support, systemic intervention, namely vaccination, was also associated with reduced psychological burden in HCWs [9].

So far, guidelines regarding the preservation and enhancement of HCWs’ well-being often lack a solid empirical foundation and tend to neglect the specific needs of the target group [10]. As the effectiveness of psychological interventions partly depends on the acceptance and attendance of the target group, factors that might be hindering HCWs need to be considered. Results from previous studies show that a lack of understanding regarding the needs of HCWs, their attitudes and scarce knowledge about the interventions as well as a lack of motivation, insufficient planning and preparedness along with a lack of time and resources were among the most common barriers to the implementation of supporting interventions for HCWs [11].

In general, qualitative investigations are vital to identify previously unknown factors [12, 13] and to identify the backgrounds and specific resources contributing to the well-being and successful coping of HCWs. So far, there has been little qualitative research investigating HCWs’ experiences, coping strategies, desires, needs and obstacles during the pandemic [14, 15]. Existing qualitative studies have mainly focused on HCWs’ general views and challenges and have neglected potential differences between occupational groups among HCWs’, such as nurses and physicians [14, 15]. Therefore, the aim of the present study was to gain elaborate insight into factors that have an impact on the management of the current challenges, as well as into those that have an inhibiting effect on the use of support services.

## Material and methods

### Study recruitment and sample

Employees of a large, urban, tertiary care center in Germany (University Hospital Augsburg, UKA) were recruited via e-mail sent to a mailing list of employees and by means of snowball sampling. The UKA is the only tertiary care hospital in the region of Bavarian Swabia with more than 1700 beds and 5000 employees. In this tertiary care hospital, all types of diseases are diagnosed and treated and the hospital owner is Bavaria (governmental ownership). Potential interviewees contacted our team as response to the invitation mail if they were interested in participating. In the recruitment e-mail, staff were informed that the interviews were conducted with the aim of gaining insights into coping strategies during the pandemic as well as desired support services to derive implications for the management of future events of a similar nature. Participants were eligible if they were employed at said tertiary care center and were clinically active. In selecting the participants, deliberate attention was paid to heterogeneity in terms of age, gender, occupation, and professional status to obtain the broadest possible spectrum of opinions and views. For this purpose, these relevant characteristics were defined in advance and requested from the interested parties before the interviews were conducted. The interviewees did not receive any compensation for their participation. Furthermore, the obtained material was analyzed concurrently with conducting the interviews and the recruitment process was ceased when the point of theoretical saturation [16] was reached. All procedures were approved by the responsible ethics committee (Ref. 20–1084) and interviews were conducted after giving written informed consent.

### Data collection and analysis

We performed semi-structured individual interviews that took place from April 8^th^ to May 6^th^, 2021, via video conference. Basic sociodemographic and professional characteristics were assessed through self-report. The interview guide (see Table 1) was developed collaboratively by the research team and required minimal revision after two pilot interviews. Key aspects were addressed using open-ended questions and examined in greater depth by means of targeted follow-up questions. All interviews were conducted by the same person. At the beginning of each interview, the interviewer introduced herself as a research assistant. There were no prior acquaintances between the interviewer and any of the participants. The recorded audio files were transcribed and anonymized by qualified professionals of a transcription office. Further, the transcripts were randomly checked for accuracy using the audio files. This process was approved by the responsible ethics committees. The statements quoted in this work have been translated into English by the interviewer (TH).

**Table 1:** Interview guide used for semi-structured interviews

**Preliminary information**	• Greeting and Introduction • Depiction of the interview topic • General information about course and duration of interview • Information about release and use of data
**Opening question**	• “We have been living with the COVID-19 pandemic for over a year now. For you personally, when was the worst time?”
**Topic Sect. 1: Changes since the beginning of the pandemic**	• “Has your professional everyday life changed since the beginning of the pandemic?” ◦ “What causes particular burdens and stress for you?” • “Have your private relationships changed since the beginning of the pandemic?” ◦ “[…] concerning family, partner, friends” ◦ “[…] concerning child care” • “Do these changes affect your personal well-being” If yes, how?” ◦ e.g. stress, sleeping problems, anxiety, helplessness
**Topic Sect. 2: Coping strategies and protective factors**	• “What helps you master your everyday work life and deal with negative events?” ◦ “How do you deal with stress? Which strategies are helpful?” ◦ “What provides a balance to work for you? Are there any resources in particular that give you strength?” ◦ “What do you do for relaxation?” ◦ “What’s your colleagues’ and superiors’ roles in dealing with the present challenges?” ◦ “Have you adopted any bad habits that help you relax?”
**Topic Sect. 3: Interventions and potential barriers**	• “What’s your opinion on the existing offers and support services that have been developed to help you cope with the current challenges? What are your experiences?” ◦ “Do you know which support services currently exist?” ◦ “Have you accessed any of these offers? What are your experiences?” ◦ “What could be the reasons for not participating in these support services?” ◦ “What offers and support services do you need or wish for that support you in your everyday work life?” ◦ “How could the working conditions in general be improved?”
**Closing question**	“Compared to the first wave of the pandemic: Have the working conditions and support services changed in any way?”
**Summary**	• Summary of the key statements by the interviewer

Twelve nurses and nine physicians varying in age, professional status and frequency of contact with COVID-19 patients took part in the interviews (see Table 2). Additionally, two members of administrative staff participated, but were excluded from further analysis due to lack of experience with challenges of daily clinical practice. Further, four interested individuals contacted the study team, but did not participate due to lack of time. Analyses focused on the complete sample and exploratory comparisons between groups were performed afterwards. The interviews lasted between ten and fifty-two minutes. At the end of each interview, member checking was performed by summarizing the interviewee’s statements and questioning the participant to determine accuracy. Suggested corrections made by the participants were recorded and considered in the following analysis steps.

**Table 2:** Sociodemographic and professional characteristics of participants (*N* = 21)

Characteristic	Physicians *n* = 9	Nursing staff *n* = 12
Sex (Female/Male)	4/5	9/3
*Age group*		
20–35 years	2	5
36–50 years	6	4
> 50 years	1	3
*Professional status*		
Leading position	8	1
Assistant leading position	0	3
No leading position	1	8
*Contact frequency with patients infected with COVID-19 since November 2020*		
Rarely	2	0
Occasionally	3	2
Frequently	4	10

**Fig. 1:**
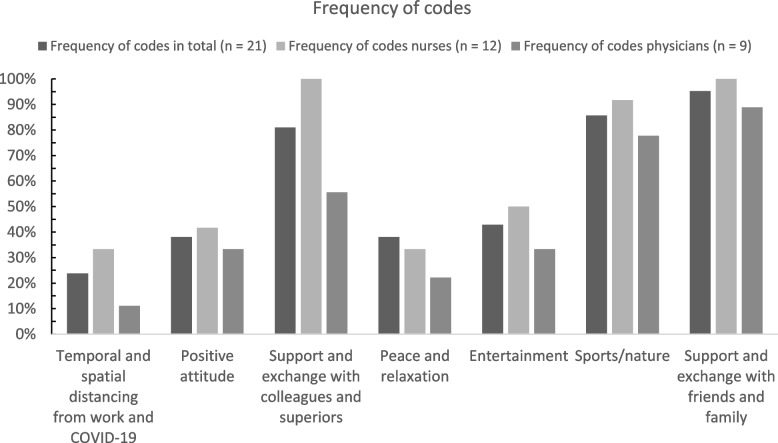
Coping strategies and protective factors. Percentages of participants who made at least one statement assigned to the respective code in the domain (main code) “Coping strategies and protective factors” in total and by profession

**Fig. 2:**
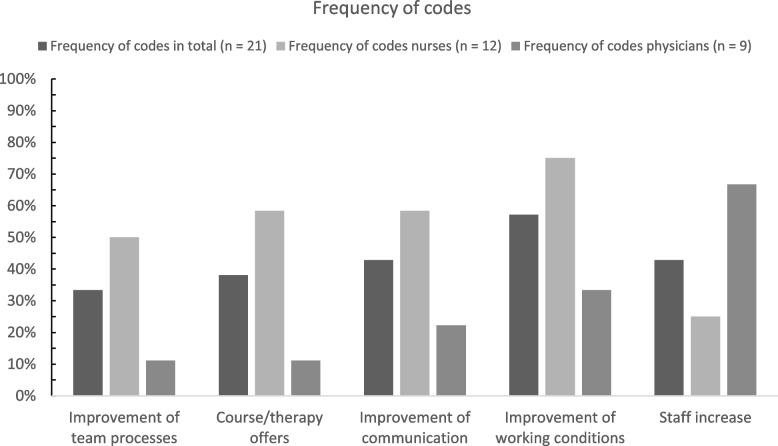
Needs and wishes. Percentages of participants who made at least one statement assigned to the respective code in the domain (main code) “Needs and wishes” in total and by profession

**Fig. 3:**
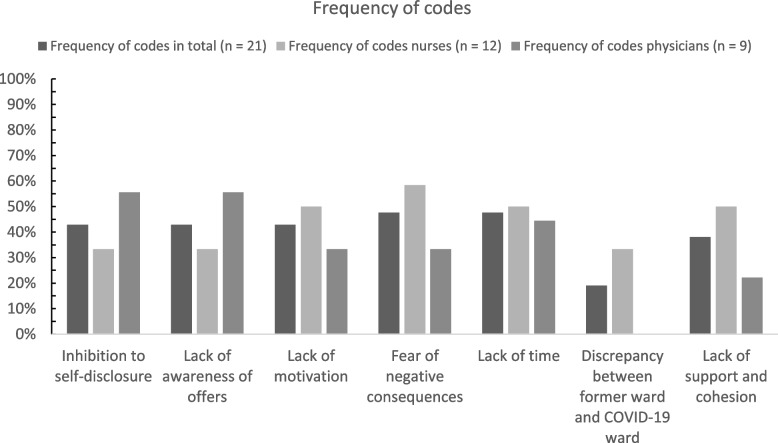
Barriers in implementing support. Percentages of participants who made at least one statement assigned to the respective code in the domain (main code) “Barriers in implementing support” in total and by profession

We analyzed the transcripts following the rules of qualitative structuring content analysis according to Kuckartz [17]. The transcripts were first checked to determine all relevant text passages and then divided into the following deductively developed main codes (codes level 1) based on the interview guide: 1. coping strategies and protective factors, 2. wishes and needs, 3. barriers in implementing support. Code systems have a hierarchical structure consisting of main codes, subcodes and subcodes of subcodes. Further codes emerged inductively while analyzing the transcripts and derived from the research questions. Subsequently, all relevant text passages were assigned to the inductively developed subcodes (codes level 2) and sorted into inductive subcodes of these codes (codes level 3) during further analyses. The entire transcripts were then categorized according to the established code system and the main and subcodes were once more modified in case of thematic overlaps. Finally, the codes were summarized and depicted using charts (see Figs. 1, 2 and 3). Analyses were conducted using the software MAXQDA Plus (release 20.4.2).

To assess the quality of the codes, two members of the research team (MS and TH) independently coded all transcripts based on the principle of consensual coding [17]. Ambiguities and remarks were noted by means of memos and any deviating coding was discussed and adjusted as necessary.

## Results

The main codes “Coping strategies and protective factors”, “Needs and wishes” and “Barriers in implementing support” were each divided into subcodes as a result of the participants’ statements. A total of 16 subcodes (see Figs. 1, 2 and 3) emerged inductively from the interviews. At the time of the interviews, limited psychological support services were offered to the teams of the participants including team supervision and individual counseling as well as access to pastoral care service, de-escalation management and psychological crisis or group interventions. Additionally, free access to professional psychiatric-psychotherapeutic services was offered and an anonymous telephone helpline was provided. Yoga and mindfulness training, for instance, were offered as recreational activities. While the majority of services are offered on a continuous basis, the pastoral counseling service was only available from February to April 2021. The interview questions on barriers in implementing support specifically refer to the aforementioned support services that were available at the time of the interviews.

### Coping strategies and protective factors

This domain, on the one hand, comprises the coping strategies that were found to be particularly helpful in dealing with the challenges and problems amplified by the SARS-CoV-2 pandemic. On the other hand, this domain includes protective factors representing attributes that have a positive impact on the well-being as well as reduce the effects of stress (see Fig. 1 for the frequency of codes).

Staff continued to seek balance in existing coping strategies, such as the use of entertainment as well as exercise and spending time outdoors. Further, special emphasis was placed on social exchange and support, on the one hand in the private sphere through family and friends, and on the other hand in a professional context through colleagues and superiors. While all interview participants from the group of the nurses stated that peer support was a helpful coping strategy, this aspect was only mentioned by five out of the nine interviewed physicians and therefore appeared to be less prominent amongst this group.

“[…] that’s how I draw most of my strength, of course, from a good family life. And that’s why, like I said earlier, I have the luxury of [having a family] compared to someone without a family, for example, who lives alone. Or also the fact that you have […] children is a luxury in a way.” (Example from Physician 7)

While support through private interactions was described as mostly unrelated to the burden of work, sharing the problems exacerbated by the pandemic with like-minded colleagues was a key coping strategy. This was explained by some respondents as a result of feeling better understood by colleagues and being able to give each other advice when needed.

“And because of that, because you realize that the other person feels the same way, you can simply cope with this stress or these difficult fates much better than if you don’t talk about it. So this conversation with colleagues who are on the same wavelength and experience the same thing, that helps me a lot.” (Example from Nurse 4)

In contrast to addressing the burdens at work, some of the interviewees felt that spatial and temporal distancing from work or any pandemic related topic was an important aspect of balance. Frontline HCWs often expressed a desire to return to their former ward to escape the stressors faced on the COVID-19 ward and to rejoin their familiar colleagues. Others described that the only possible way to relax after work was maintaining clear boundaries regarding working hours.

“Yes, so now I draw strength from the fact that I can leave the ward again […] (laughing) and I can be replaced there […], because I have asked to be replaced there by someone else. And that actually gives me strength, because that’s a time that you can bridge.” (Example from Nurse 4)

Being able to draw strength and maintain a positive attitude was seen by some as fundamental protective factors in overcoming the challenges at hand. These abilities were mostly attributed to one’s character traits, such as an optimistic and humorous personality.

### Needs and wishes

This domain addresses the needs and wishes of the HCWs, which could serve as support in everyday work and in coping with the prevailing strains and burdens (see Fig. 2 for the frequency of codes).

Staff’s responses about their needs and ideas for improvement were divided, especially when comparing nurses’ and physicians’ answers. Whilst nurses perceived opportunities for optimization primarily at an in-hospital level, such as through the improvement of working conditions and communication processes, the surveyed physicians considered the greatest opportunity for optimization to be an increase in staff.

“Yes, more staff. I believe that in nursing in particular, even without corona, people often wish there was more, more manpower, to support everything. Also to have more time with the patient, which is simply also necessary to actually care [for patients], right?” (Example from Physician 6)

The need for practice-oriented improvements of working conditions in general, such as better planning of staff work schedules, flatter organizational structures and prompt assistance with problems, was particularly emphasized by nurses. Some interviewees described the impression that their concerns were invalidated and not being taken seriously by decision-makers, including both immediate superiors as well as higher-level executives.

“[…] I would have liked to have someone help me when I said something and there is a need, that when something happens that you don’t just have the feeling that everything always disappears into thin air. For example, it would have been very important for me to have someone respond to my workload complaint letter.” (Example from Nurse 1)

When asked about their wishes, the need for improved communication processes, both within and between teams, was frequently talked about by the interviewed nurses. The need for the development of an error culture, that promotes an open, non-judgmental approach to errors,was further addressed by some employees, which has so far been neglected in the hospital system. In addition to improving communication, an important wish for many was to improve team processes and team spirit, in particular through gestures of mutual appreciation.

“[…] so primarily, I think, communication among each other […] I think I would like to see fixed times and rules in the team. So especially with regard to the handover, that it is simply obligatory to say that there will now be a verbal handover. Simply that one speaks a few more words with each other and less [information] is lost.” (Example from Nurse 6)

“But I think we need to learn a few things together again. I think we need to learn about the culture of making mistakes. I think we have to learn appreciation.” (Example from Physician 5)

While the workers appreciated the existing course and therapy offers, some respondents wanted more varied, low-threshold offers, such as visits by psychological staff to the wards without prior appointments. An extension of supervision sessions, in terms of frequency and participation rate,was particularly asked for, with some respondents considering an obligatory participation necessary.

### Barriers in implementing support

Due to various barriers, existing support services offered by the participants’ employer were often not accessed. Furthermore, the participants reported negative influencing factors that had a detrimental effect on their daily work life in general (see Fig. 3 for the frequency of codes).

Although most nurses and physicians perceived peer support as one of the most helpful coping strategies, some staff described a lack of cohesion and exchange within their teams. This was mentioned primarily in connection with the atmosphere on the COVID-19 ward and the discrepancy between the COVID-19 ward and the interviewee’s respective former ward, which was exclusively mentioned by the interviewed nurses. Nurses reported as important stress factors, first, that the newly formed teams for COVID-19 wards were randomly formed from stable teams without respecting previous team structures and needs. Second, nurses defined the lack of a uniform standard of work regarding the execution of work tasks in these new teams as a second important stress factor.

“The new colleagues, I can’t really call them colleagues. Because they were [just] people I worked with. We never had time to really get to know each other, so to speak. […] So I only ever worked with some of them at the end, and I actually saw them maybe twice because of this jumping between the wards.” (Example from Nurse 1)

Regarding participation in course and therapy offers, there was a great variety of experiences among the surveyed staff, especially in terms of awareness of the existence as well as the benefits of offered services. While most participants felt that offers were well advertised, others were unaware of the available support and its potential benefits. Other employees expressed that while they were aware of the existence of such offers, they were uncertain about whether support services could be beneficial for them in terms of coping with the challenges and burdens.

“[…] that you don’t see the awareness or the chance that through such an offer, or that things could get better. Wanting to sort it out with yourself is, I think, a hindrance.” (Example from Physician 7)

Further, one frequently reported barrier was the lack of free time among the workers, which many of the participants explained as being due to increased workloads, numerous hours of overtime and shift work that prevents regular attendance at fixed appointments. Simultaneously, several respondents mentioned a lack of motivation as a possible inhibiting factor, as the limited free time and the high workload resulted in a reduced interest in participating in activities after the end of a workday.

“I also believe that because of the workload, now the pure timely workload, many employees in the house are also very happy when they just finish their shift and clock out and go home. Because then it’s over and done with, they leave and go home and that’s it.” (Example from Physician 4)

Additionally, several participants in both groups stated that the fear of opening up and the disclosure of personal, intimate information was a major barrier to seeking psychological support services. On the one hand, some employees expressed concern about admitting and realizing their own mistakes and alleged weaknesses and subsequently dealing with them; on the other hand, they described that the confrontation with burdening or traumatizing experiences might only complicate and prolong the process of moving on. Some interviewees seemed to hold the perception that everyone must cope with the stressors and strains themselves and that it was a matter of “getting a hold on yourself”.

“And that [seeking help] is certainly the more strenuous way, it leads to feeling and having feelings in this profession is perhaps more—yes, it is simply more strenuous.” (Example from Nurse 6)

“[…] I’m sure that there are some outdated role models that suggest: This shouldn’t affect me so deeply; after all, I am—and they wouldn’t consider such offers in order not to show any weakness.” (Example from Physician 5)

In addition, several of the employees feared negative consequences as a result of seeking psychological support services. This aspect was more dominant among the group of nurses compared the interviewed physicians. Workers were particularly concerned about the potential lack of anonymity within the hospital and any resulting disadvantages, as well as the fear of being stigmatized by their colleagues and superiors.

“[…] you will be stigmatized a bit. If I were to seek psychological help now, I would keep that to myself. This is a hard business, without criticizing anyone here, but the psychological care after a covid infection or any other psychological support; that stigmatizes you, I must say that quite clearly. […] but in our tough business, where the demands are quite high, you are quickly stigmatized or somehow seen differently.” (Example from Physician 8)

In line with the aforementioned fear of acknowledging one’s own need for support, several of the workers described their main fear as being perceived as weak or mentally unstable by their colleagues and thus making themselves vulnerable.

“I think that a lot of people are constantly afraid that some of their weaknesses or mistakes will come to light and that this will make them more vulnerable.” (Example from Nurse 11)

Further, the presence of colleagues during supervision sessions in team settings was an obstacle for some workers, who feared possible interpersonal conflicts because of expressing their opinions and thoughts.

## Discussion

The aim of this study was to gain a deeper understanding of HCWs’ coping strategies, protective factors, wishes and needs as well as potential barriers preventing them from participating in support services offered by their employer in times of the SARS-CoV-2 pandemic. Regarding certain aspects, there was considerable consensus among the interviewees and prevailing trends could be identified. The importance of addressing mental health issues among HCWs has been particularly highlighted during the pandemic as a result of the consequences that may arise from increases in workload, traumatic experiences and unaddressed mental health problems [18]. These challenges and adverse working conditions, which have been exacerbated by the pandemic, pose risk factors for HCWs’ turnover intention worldwide, which in turn aggravates problems such as shortage of manpower as well as increased workloads for remaining HCWs [18].

With regard to existing protective factors, peer and family support were defined as key factors. While talking to peers was greatly valued due to being able to share the experienced burdens with like-minded people, spending time with family and friends was seen as a way of relaxation and distancing oneself from work. These results are in line with other studies emphasizing the importance of support from family, friends or colleagues [19] for coping with the consequences of the pandemic. In addition, previous research provides evidence that the results of our study may be applicable to other professional groups in the medical field besides nurses and physicians. For instance, one systematic review and a survey involving 168 behavioral health staff conducted in the United States showed that social support from family, friends, supervisors and colleagues was a key coping strategy not only for nurses and physicians, but also for other medical professionals such as midwives, radiologists, physiotherapists, pharmacists [8] as well as behavioral health clinical and administrative staff [20]. Additional coping strategies that were identified as particularly helpful for several professional groups in the medical field included individual and group counseling with mental health professionals as well as effective leadership [8]. Aside from healthcare professionals, a scoping review found that peer support and an optimistic attitude were also key coping strategies for nursing students [21]. These findings highlight the relevance of peer support across multiple professions and provide important indications for increasing the focus on peer support in future support services.

While the majority of the interviewed employees appeared to be confident in being able to cope with the events they had experienced on their own, a recent meta-analysis including 132 studies showed that post-traumatic stress disorder (PTSD) was very common during the SARS-CoV-2 pandemic in that population [22]. Considering the results from another meta-analysis including 24 studies, which showed that approximately 25% of PTSD cases had an onset of symptoms at least 6 months after the stressor [23], it can be assumed that nurses’ and physicians’ long-term well-being may benefit from monitoring their mental health and the access to psychological support services beyond the period of experienced stress. The aforementioned meta-regression findings further provided evidence that prevalence rates across all aforementioned mental health disorders were higher among frontline HCWs than general HCWs [22]. Similarly, our previous survey showed that the levels of stress, exhaustion and depressive mood were higher and levels of work-related job-fulfilment were lower among nurses working on COVID-19 wards than those of nurses working on regular wards [6].

Among the barriers which make the use of support services less likely, stigmatization from colleagues, supervisors or the public as well as self-stigmatization was a recurring topic among our sample. This fear of stigmatization and negative consequences can likely also be seen as a culmination of the fear of making mistakes, the prevailing expectation to perform and the resulting shame when errors do occur as well as the work-related tradition of showing no weakness, as mentioned by several interviewees. Similar conclusions can be drawn from a cross-sectional study on the barriers to seeking help for mental health issues among a sample of 98 HCWs [24]. The findings showed that the predominant barriers to seeking help were the worry about exposure among colleagues as well as the fear of negative consequences [24]. In view of these potentially negative effects, it may be helpful to highlight the management of errors in everyday professional life, especially with regard to the medical, ethical, societal and legal challenges when working with patients suffering from SARS-CoV-2. Moreover, responses obtained from over 1000 physicians in a survey from the United Kingdom found perceived stigma and fear of disappointing colleagues by admitting to mental health problems to lead to a reduced use of psychological support services [25]. The fear of stigmatization and showing vulnerability might therefore be a key barrier to seeking psychological help. Underlying causes of this fear of stigmatization may lie in the prevailing organizational culture. Assumptions and prejudices that may explain the processes of stigmatization in work environments about individuals who seek psychological help include the belief that they are unable to meet the demands of their work tasks and that they pose a certain danger and unpredictability [26]. In the occupational context, doubts about the legitimacy of mental illnesses may present an additional factor of stigmatization, which imputes that those affected are feigning an illness in order to avoid work [26]. However, one should bear in mind that during the pandemic, HCWs were under severe pressure, so that the reported fear of making mistakes must be discussed in that context. In Germany, many rules and laws regarding the management of patients with SARS-CoV-2 were made and HCWs were under constant pressure of publicity – those factors may have led to a certain level of uncertainty and fear by HCWs. In contrast to the working environments before the pandemic, the new pandemic situation may have fostered the reported fears.

Regarding needs and suggestions for improvement, there was a considerable disparity between the two professional groups. While the nurses saw opportunities for improvement mainly in practice-related and organizational matters, such as improving communication and the flow of information, physicians generally expressed few ideas for improvement other than the need for an increase in staff. Noticeable differences between nurses and physicians have also been identified in the prevalence of mental health issues, as reported in a recently published umbrella review including 44 meta-analyses [27]. It was shown that the prevalence rates of anxiety, depressive symptoms, insomnia and sleep disturbances were higher in nurses than in physicians, while the physicians’ group showed higher prevalence rates regarding stress as well as symptoms of post-traumatic stress [27]. These possible differences in the professional groups’ mental health may contribute to the discrepancy between the statements reported in the present study. With regard to the differences between the two occupational groups, gender-specific factors should also be taken into account. One meta-analysis of 117 studies showed that female HCWs presented higher prevalence of PTSD and anxiety during the SARS-CoV-2 pandemic, whereas no gender differences were reported for stress, depression and burnout [28]. Considering the imbalanced gender ratio in the group of interviewed nurses in our study, in which the proportion of female participants was 75%, gender differences may be an additional factor influencing the results of our study. Further, one meta-analysis of 33 studies with a total of 31,071 participants identified several factors that are related to psychological resilience, which may mitigate stress, allow an individual to adapt to stressful or traumatic events [29] and may further contribute to the diversity of statements observed in our study. Sociodemographic factors associated with resilience were age and gender, while protective factors included life satisfaction, optimism and social support [29]. Moreover, there are a number of other factors (e.g. working environment, time resources, and individual gratification) that need to be taken into account that could determine the different needs of the professional groups.

### Limitations

When interpreting the findings of the present study, one needs to consider that all interviews were conducted during a particular time point of the pandemic and may therefore not reflect the entirety of challenges experienced by HCWs throughout the pandemic as a whole. Further, exclusively nurses and physicians were included in our study and no conclusions regarding other healthcare professionals can be drawn. In addition, the occurrence of recall bias in the sense of memory errors and social desirability bias cannot be ruled out, since several interview questions addressed sensitive topics (e.g. harmful coping strategies) as well as past experiences. As a further limitation, one should note that detailed sociodemographic information and the frequency of individual contacts with SARS-CoV-2 patients were not assessed. Moreover, participants were not selected randomly so that we cannot exclude a sampling bias (e.g. that mainly affected HCWs participated). The reason was that due to the workload during the pandemic and the content of the topic, we had difficulties to recruit more HCWs than needed. However, the planned sample size and theoretical saturation point were reached and are comparable to other qualitative studies. Since qualitative research does not intend to obtain the largest possible number of cases and thus does not intend to achieve representativeness by means of statistically standardized research methods [12], the results of the present study did not have the aim to achieve generalizability of the results, but to detect novel aspects of psychosocial stress in HCWs during the pandemic.

### Conclusions

Our findings suggest that future support offers should place greater emphasis on peer support in mixed professional groups. The analysis indicates that the impact of potential outdated role models, such as the constant pressure to perform while concealing weaknesses and a deficient expression of appreciation, requires additional investigation. A critical barrier to seeking help reported in this study is the fear of stigmatization. Promoting a more open approach to mental health problems, ensuring anonymity and addressing employees’ attitudes towards mental disorders may be potential solutions to overcome this obstacle. Further research is also needed to investigate individual, systemic and organizational barriers to the implementation of interventions and options for the improvement of workings conditions.

## Data Availability

Data supporting the findings of this study have not been made generally available due to the personal and sensitive content of the participants’ accounts. Data can be made available upon reasonable request to the corresponding author (TH).

## Abbreviations

HCWs: Healthcare workers
PPE: Personal protective equipment
PTSD: Post-traumatic stress disorder
